# Berberine Attenuated Aging-Accelerating Effect of High Temperature in Drosophila Model

**DOI:** 10.4236/ajps.2014.53037

**Published:** 2014-01-26

**Authors:** Valeriya Navrotskaya, Gregory Oxenkrug, Lyudmila Vorobyova, Paul Summergrad

**Affiliations:** 1 V. N. Karazin Kharkiv National University, Kharkiv, Ukraine; 2 Tufts University School of Medicine, Tufts Medical Center, Boston, USA

**Keywords:** Berberine, Life Span, Drosophila Melanogaster, High Temperature, Kynurenine

## Abstract

We have observed that berberine prolonged life span and improved viability of pupae and climbing activity of imagoes of wild-type Drosophila melanogaster maintained at 23°C. As a continuation of our studies of berberine effect on life span, we were interested to evaluate the effect of berberine of life span in flies maintained at a higher temperature (28°C) known to accelerate aging in wild type flies. Considering that genetically or pharmacologically induced deficiency of TRP conversion into KYN prolonged life span in a Drosophila model, we compared the effects of berberine, a powerful inhibitor of kynurenine (KYN) formation from tryptophan (TRP), on life span in wild type and in Drosophila melanogaster mutants (vermilion) with deficient TRP-KYN metabolism maintained at 23°C and 28°C. High (28°C) ambient temperature decreased life span in both wild type and vermilion flies. Aging accelerating effect of high temperature was more pronounced in Oregon than in vermilion flies (−60% vs. −40% decrease of mean life span, resp). Berberine attenuated the aging-accelerating effect of high temperature. Effect of berberine was more pronounced in Oregon (+46%) than in vermilion (+22%) flies. The obtained data suggested the possible involvement of TRP-KYN metabolism in the aging-acceleration effect of the high temperature and in protective effect of berberine.

## 1. Introduction

Up-regulation of kynurenine (KYN) pathway of tryptophan (TRP) metabolism was suggested as one of the mechanisms of neurodegenerative disorders of aging [1-3]. We found that Drosophila melanogaster mutants with deficient formation of KYN, *vermilion* and *white*, had longer life span than wild type flies (Oregon) [4]; and that inhibitors of TRP-KYN metabolism, alpha-methyl-TRP and 5-methyl-TRP, prolonged life span of wild-type flies [5]. Inhibition of TRP-KYN metabolism exerted neuroprotective effect in flies [6]. Furthermore, inhibitors of TRP-KYN metabolism available for human use, berberine, isoquinoline alkaloid isolated from *Berberis aristata*, a major herb widely used in Indian and Chinese systems of medicine, is a strong inhibitor of the rate-limiting enzyme of TRP-KYN metabolism the active ingredient of an herbal medicine [7, 13] and minocycline, an antibiotic with anti-inflammatory effects, prolonged life span and stimulated locomotor activity (negative geotaxis) of wild type flies [8-10].

As a continuation of our studies of berberine effect on life span we were interested to evaluate the effect of berberine of life span in flies maintained at a higher temperature (28°C). It is known that life span is temperature dependent, and flies are living faster at the higher temperature [11], *i.e*, there is an inverse relationship between life span and temperature [12]. Our working hypothesis was that berberine protects against the aging-accelerating effect of high ambient temperature. To check this hypothesis we compared the effect of berberine on life span of Oregon flies kept at 23°C and 28°C.

Considering that berberine is a strong inhibitor of kynurenine formation from tryptophan [13], and that genetically or pharmacologically induced deficiency of TRP conversion into KYN prolongs life span [4, 5, 14], we compared the effects of berberine on life span in wild type and KYN formation deficient mutants (*vermilion*) of *Drosophila melanogaster* maintained under 23°C and 28°C.

## 2. Methods

Wild-type stock Oregon of *Drosophila melanogaster* and mutant stock *vermilion* from the collection of V. N. Karazin Kharkiv National University were used in the experiments. The study was carried out between June and August.

Flies were maintained at 23°C in a 12:12 light: dark period on a standard *Drosophila* medium consisting of sugar, yeast, agar and semolina. Berberine (Sigma Aldrich Chemical Co, USA) was added to nutrition medium in the dose of 1 mM (0.4 mg/ml of nutrition medium) at a larvae stage. Effective doses of berberine were selected by us in a previous investigation [7].

Flies collected in control and berberine variants were divided into two groups, one of which maintained at 23°C and the other at 28°C.

Life span evaluation: one day old adult flies (males) were collected and then regularly transferred to fresh medium every 3 - 4 days. The number of dead flies was recorded at the time of transfer.

The obtained data were statistically analyzed using Wilcoxon rank-sum test and two ways ANOVA test.

## 3. Results

Effect of impaired formation of KYN on life span. Life span of *vermillion* male flies maintained under 23°C was longer (by 43%) than life span of Oregon flies in accord with our previously published data [4] (Table 1).

**Figure 1:**
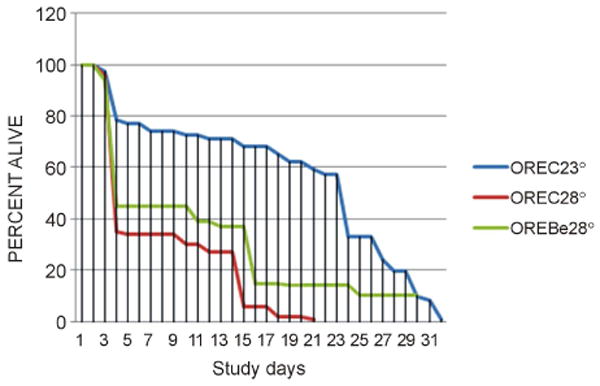
Berberine effect in Oregon flies. OREC23°: Oregon male controls flies kept at 23°C; OREC28°: Oregon male controls flies kept at 28°C; OREBe28°: Oregon male flies kept at 28°C with addition of berberine.

**Figure 2:**
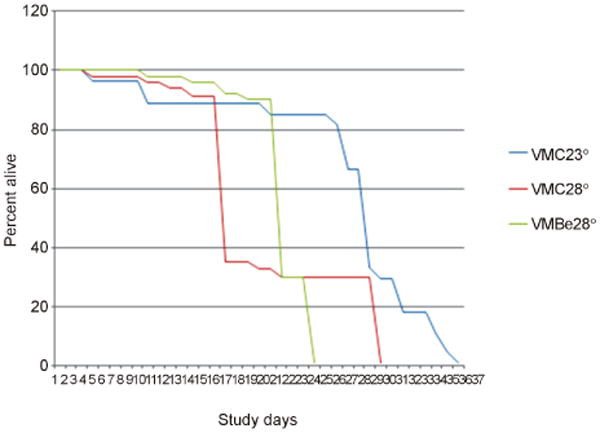
Berberine effect in vermilion flies. vmc23°: vermilion male controls flies kept at 23°C; vmc28°: vermilion male controls flies kept at 28°C; vmBe28°: vermilion male flies kept at 28°C with addition of berberine.

Effect on high ambient temperature on life span. High ambient temperature (28°C) decreased life span of Oregon flies in accordance with literature data (see above) (Figure 1). High ambient temperature decreased life span of *vermillion* flies as well (Figure 2). The effect of high temperature on life span was less pronounced in *vermilion* (−40%) than in Oregon (−60%) flies (Table 1).

Berberine and life span of flies maintained at high temperature. Berberine attenuated the effect of exposure to 28°C on life span in Oregon (Figure 1) and *vermilion* flies (Figure 2). Berberine prolonged the life span of Oregon flies by 46% in comparison with control flies kept at 28°C and by 22% in vermilion flies kept at 28°C (Table 1).

## 4. Discussion

The main finding of our study is berberine-induced attenuation of aging-accelerating effect of high temperature in Drosophila melanogaster model. We previously reported that berberine prolonged the life span of Drosophila flies kept at 23°C [7]. The present data indicate that berberine may enhance the ability of fruit flies to resist stress caused by high temperature, since its addition to larvae attenuated the aging-accelerating effect of high temperature. The free radical theory of aging hypothesizes that oxygen-derived free radicals are responsible for the age-related damage at the cellular and tissue levels [15], and at temperature-accelerated aging these processes might be especially intensive [11, 12]. So the protective effect of berberine may be dependent on its anti-oxidant activity [16].

The alternative mechanism of observed protective effect of berberine against aging-accelerating action of high temperature may be related to berberine-induced inhibition of TRP-KYN metabolism. Berberine is a stronger inhibitor of TRP conversion into KYN than a “standard” inhibitor, 1-methyl-TRP [13]. Genetic and pharmacological inhibition of TRP-KYN pathway extends life span of Drosophila. Flies with mutations *white* (impaired transmembrane transport of TRP into cells where its metabolism occurs) and *vermilion* (deficiency of rate-limiting enzyme of TRP-KYN pathway, TRP 2,3-dioxygenase) had longer life spans than wild-type flies [4, 14]. Among pharmacological inhibitors of KYN formation from TRP used in our experiments were alpha-methyl-TRP and 5-methyl-TRP, berberine and minocycline, all of them prolonged the life span of wild-type *Drosophila melanogaster* [5, 7, 8].

Berberine-induced attenuation of aging-accelerating effect of high temperature (28°C) was less pronounced in *vermilion* (22%) than in Oregon flies (46%) (Table 1) might suggest the involvement of TRP-KYN pathway in mechanisms of high temperature effect on the life span. This suggestion might be further confirmed by less pronounced protective effect of berberine against high temperature-induced acceleration of aging in *vermilion* than in wild-type flies (Table 1). Present data warrant further studies of the involvement of TRP-KYN metabolism in mechanisms of high temperature-induced acceleration of aging and protective effect of berberine and other inhibitors of TRP-KYN metabolism.

The results of present and our previous studies of berberine action on life span, viability and stress-resistance of drosophila indicate that berberine is a potentially good candidate drug for anti-aging intervention and attenuation of stressful conditions impact.

## Figures and Tables

**Table 1:** Life span at different temperatures and effect of berberine, in drosophila stocks

Stock	Experimental groups		

	23°C	28°C	28°C + berberine
Oregon	19.94 ± 1.24* n = 168	7.85 ± 0.64 n = 150	11.51 ± 1.35** n = 98
*Vermilion*	28.52 ± 1.65* n = 100	17.36 ± 0.57 n = 94	21.19 ± 0.43# n = 92

* Mean ± standard error; n = number of flies; P = 0.001 vs. 28°C and 28°C + berberine;

** P = 0.001 vs. 28°C;

# P = 0.001 vs. 28°C.
